# (1*S*,2*S*,6*S*,9*S*)-6-Methyl-5-oxobicyclo­[4.4.0]decane-2,9-diyl diacetate

**DOI:** 10.1107/S1600536810036639

**Published:** 2010-09-18

**Authors:** Riina Aav, Kristin Lippur, Margus Lopp, Franz Werner

**Affiliations:** a Tallinn University of Technology, Department of Chemistry, Akadeemia tee 15, 12618 Tallinn, Estonia

## Abstract

The chiral title compound, C_15_H_22_O_5_, is an inter­mediate in the total synthesis of biologically active 9,11-secosterols. In the crystal, the cyclo­hexane rings are *trans*-fused and both adopt chair conformations. In the crystal, mol­ecules are loosely held together in a layer parallel to (100) by weak inter­molcular C—H⋯O hydrogen bonds accepted by carbonyl O atoms of the acetyl groups.

## Related literature

For background to the biological activity of 9,11-secosterols and the synthesis of the title compound, see: Aav *et al.* (2000 ▶). For a related structure, see: Foot *et al.* (2006 ▶). For hydrogen bonding, see: Steiner (2002 ▶). 
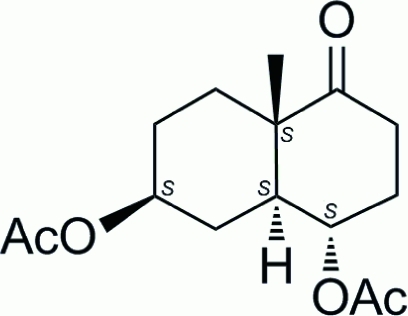


## Experimental

### 

#### Crystal data

C_15_H_22_O_5_
*M* *_r_* = 282.33
Monoclinic, 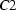

*a* = 22.885 (5) Å
*b* = 9.340 (2) Å
*c* = 7.2250 (13) Å
β = 101.280 (6)°
*V* = 1514.5 (5) Å^3^
*Z* = 4
Mo *K*α radiation
μ = 0.09 mm^−1^
*T* = 300 K
0.50 × 0.20 × 0.16 mm

#### Data collection

Bruker SMART X2S benchtop diffractometer
Absorption correction: multi-scan (*SADABS*; Sheldrick, 2008*b* ▶) *T*_min_ = 0.955, *T*_max_ = 0.985
4796 measured reflections
1413 independent reflections
1226 reflections with *I* > 2σ(*I*)
*R*_int_ = 0.041

#### Refinement

*R*[*F* ^2^ > 2σ(*F* ^2^)] = 0.037
*wR*(*F* ^2^) = 0.099
*S* = 1.05
1413 reflections
185 parameters
1 restraint
H-atom parameters constrained
Δρ_max_ = 0.15 e Å^−3^
Δρ_min_ = −0.14 e Å^−3^

### 

Data collection: *GIS* (Bruker, 2010 ▶); cell refinement: *APEX2* (Bruker, 2010 ▶) and *SAINT* (Bruker, 2009 ▶); data reduction: *SAINT*; program(s) used to solve structure: *SHELXS97* (Sheldrick, 2008*a* ▶); program(s) used to refine structure: *SHELXL97* (Sheldrick, 2008*a* ▶); molecular graphics: *Mercury* (Macrae *et al.*, 2006 ▶); software used to prepare material for publication: *SHELXL97*.

## Supplementary Material

Crystal structure: contains datablocks global, I. DOI: 10.1107/S1600536810036639/is2600sup1.cif

Structure factors: contains datablocks I. DOI: 10.1107/S1600536810036639/is2600Isup2.hkl

Additional supplementary materials:  crystallographic information; 3D view; checkCIF report

## Figures and Tables

**Table 1:** Hydrogen-bond geometry (Å, °)

*D*—H⋯*A*	*D*—H	H⋯*A*	*D*⋯*A*	*D*—H⋯*A*
C8—H8*B*⋯O3^i^	0.97	2.62	3.551 (4)	161
C13—H13*C*⋯O3^ii^	0.96	2.63	3.533 (4)	156
C8—H8*A*⋯O5^iii^	0.97	2.44	3.309 (4)	149
C11—H11*A*⋯O5^iii^	0.96	2.70	3.662 (4)	178

Symmetry codes: (i) 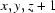; (ii) 
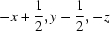
; (iii) 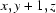.

**Fig. 1.**
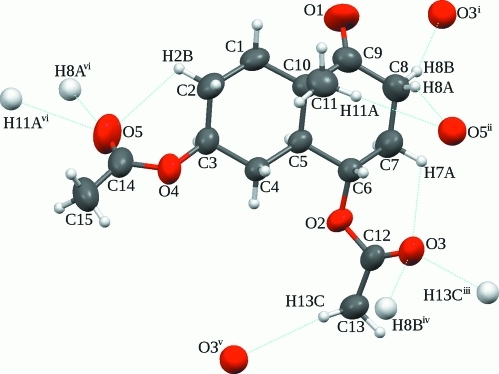
Asymmetric unit in the crystal structure of (I). Displacement ellipsoids for non-H atoms are drawn at the 50% probability level. Cyan dashed lines indicate weak hydrogen bonds. [Symmetry codes: (i) x, y, 1 + z; (ii) x, 1 + y, z; (iii) 1/2 - x, 1/2 + y, -z; (iv) x, y, -1 + z; (v) 1/2 - x, -1/2 + y, -z; (vi) x, -1 + y, z.]

**Fig. 2.**
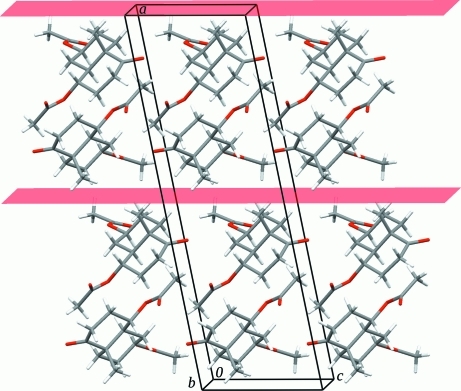
Packing diagram of (I). Red planes indicate the boundaries of the layers within which weak hydrogen bonds occur. The unit cell is outlined.

## supplementary crystallographic information

### Comment

At 300 K the enantiopure compound (1*S*,2*S*,6*S*,9*S*)-6-Methyl-5-oxobicyclo[4.4.0]decane-2,\ 9-diyl diacetate, (I), crystallizes in the chirodescriptive monoclinic space group *C*2 (No. 5) with one molecule in the asymmetric unit. Bond lengths and bond angles in the molecule are normal. The *trans*-fused cyclohexane rings both adopt chair conformation. The acetyl groups are inclined to the least-squares plane, defined by the carbon atoms of the cyclohexane rings, by ~46.4 (O2O3C12C13) and ~51.2° (O4O5C14C15), respectively (Fig. 1). The molecules are loosely hold together in layers parallel to the *A*-plane with a repeating distance of *d*_100_/2~11.2 Å, within which weak intra- and intermolecular hydrogen bonds (Steiner, 2002) occur (Fig, 2, Table 1). Between the layers only hydrophobic interactions are present.

### Experimental

Enantiopure (I) was synthesized according to Aav *et al.* (2000). Single crystals were grown by slow evaporation of a solution of (I) in acetone/petrol ether.

### Refinement

Owing to absence of significant anomalous scattering, Friedel pairs were merged and all *f*'' values were set to zero for the final refinement. The absolute structure was assigned from the synthetic procedure. Hydrogen atoms were included at calculated positions [*d*(C—H) = 0.96 (CH_3_), 0.97 (CH_2_) or 0.98 Å (CH)] and treated as riding on their base atoms, with *U*_iso_(H) = 1.2*U*_eq_(C) (CH_2_ and CH) or 1.5*U*_eq_(C) (CH_3_).

### Crystal data

**Table:**

C_15_H_22_O_5_	*F*(000) = 608
*M**_r_* = 282.33	*D*_x_ = 1.238 Mg m^−^^3^
Monoclinic, *C*2	Mo *K*α radiation, λ = 0.71073 Å
Hall symbol: C 2y	Cell parameters from 2044 reflections
*a* = 22.885 (5) Å	θ = 2.4–23.9°
*b* = 9.340 (2) Å	µ = 0.09 mm^−^^1^
*c* = 7.2250 (13) Å	*T* = 300 K
β = 101.280 (6)°	Needle, colorless
*V* = 1514.5 (5) Å^3^	0.50 × 0.20 × 0.16 mm
*Z* = 4	

### Data collection

**Table:**

Bruker SMART X2S benchtop diffractometer	1413 independent reflections
Radiation source: XOS X-beam microfocus source	1226 reflections with *I* > 2σ(*I*)
doubly curved silicon crystal	*R*_int_ = 0.041
ω scans	θ_max_ = 25.0°, θ_min_ = 2.4°
Absorption correction: multi-scan (*SADABS*; Sheldrick, 2008*b*)	*h* = −27→27
*T*_min_ = 0.955, *T*_max_ = 0.985	*k* = −11→11
4796 measured reflections	*l* = −7→8

### Refinement

**Table:**

Refinement on *F*^2^	Secondary atom site location: difference Fourier map
Least-squares matrix: full	Hydrogen site location: inferred from neighbouring sites
*R*[*F*^2^ > 2σ(*F*^2^)] = 0.037	H-atom parameters constrained
*wR*(*F*^2^) = 0.099	*w* = 1/[σ^2^(*F*_o_^2^) + (0.0528*P*)^2^ + 0.0812*P*] where *P* = (*F*_o_^2^ + 2*F*_c_^2^)/3
*S* = 1.05	(Δ/σ)_max_ < 0.001
1413 reflections	Δρ_max_ = 0.15 e Å^−^^3^
185 parameters	Δρ_min_ = −0.14 e Å^−^^3^
1 restraint	Extinction correction: *SHELXL97* (Sheldrick, 2008a), Fc^*^=kFc[1+0.001xFc^2^λ^3^/sin(2θ)]^-1/4^
Primary atom site location: structure-invariant direct methods	Extinction coefficient: 0.010 (2)

### Special details

**Table:**

Geometry. All e.s.d.'s (except the e.s.d. in the dihedral angle between two l.s. planes) are estimated using the full covariance matrix. The cell e.s.d.'s are taken into account individually in the estimation of e.s.d.'s in distances, angles and torsion angles; correlations between e.s.d.'s in cell parameters are only used when they are defined by crystal symmetry. An approximate (isotropic) treatment of cell e.s.d.'s is used for estimating e.s.d.'s involving l.s. planes.
Refinement. Refinement of *F*^2^ against ALL reflections. The weighted *R*-factor *wR* and goodness of fit *S* are based on *F*^2^, conventional *R*-factors *R* are based on *F*, with *F* set to zero for negative *F*^2^. The threshold expression of *F*^2^ > σ(*F*^2^) is used only for calculating *R*-factors(gt) *etc*. and is not relevant to the choice of reflections for refinement. *R*-factors based on *F*^2^ are statistically about twice as large as those based on *F*, and *R*- factors based on ALL data will be even larger.

### Fractional atomic coordinates and isotropic or equivalent isotropic displacement parameters (Å^2^)

**Table:**

	*x*	*y*	*z*	*U*_iso_*/*U*_eq_	
C1	0.41782 (12)	0.1268 (3)	0.8403 (4)	0.0548 (7)	
H1A	0.4505	0.1429	0.9460	0.066*	
H1B	0.3854	0.0837	0.8891	0.066*	
C2	0.43814 (13)	0.0228 (3)	0.7031 (5)	0.0603 (8)	
H2A	0.4735	0.0603	0.6649	0.072*	
H2B	0.4484	−0.0683	0.7656	0.072*	
C3	0.38967 (12)	0.0004 (3)	0.5307 (4)	0.0507 (7)	
H3	0.3559	−0.0484	0.5683	0.061*	
C4	0.36824 (12)	0.1415 (3)	0.4352 (4)	0.0481 (6)	
H4B	0.3359	0.1237	0.3292	0.058*	
H4A	0.4005	0.1868	0.3878	0.058*	
C5	0.34678 (11)	0.2411 (2)	0.5769 (4)	0.0417 (6)	
H5	0.3154	0.1895	0.6241	0.050*	
C6	0.31877 (12)	0.3794 (3)	0.4877 (4)	0.0460 (6)	
H6	0.3485	0.4372	0.4405	0.055*	
C7	0.29180 (14)	0.4638 (3)	0.6307 (4)	0.0619 (8)	
H7B	0.2590	0.4099	0.6630	0.074*	
H7A	0.2761	0.5536	0.5745	0.074*	
C8	0.33767 (15)	0.4945 (3)	0.8099 (4)	0.0625 (8)	
H8A	0.3653	0.5665	0.7822	0.075*	
H8B	0.3174	0.5338	0.9044	0.075*	
C9	0.37226 (13)	0.3646 (3)	0.8900 (4)	0.0559 (7)	
O1	0.38133 (13)	0.3386 (3)	1.0571 (3)	0.0894 (8)	
C10	0.39716 (11)	0.2710 (3)	0.7505 (4)	0.0458 (6)	
C11	0.45030 (12)	0.3532 (4)	0.6993 (4)	0.0619 (7)	
H11A	0.4377	0.4477	0.6568	0.093*	
H11B	0.4645	0.3033	0.6006	0.093*	
H11C	0.4817	0.3598	0.8085	0.093*	
O2	0.27152 (7)	0.33569 (19)	0.3333 (2)	0.0510 (5)	
C12	0.25230 (13)	0.4324 (3)	0.1969 (4)	0.0523 (7)	
O3	0.26894 (10)	0.5536 (2)	0.2034 (3)	0.0678 (6)	
C13	0.20704 (15)	0.3674 (4)	0.0446 (4)	0.0689 (9)	
H13A	0.1906	0.4400	−0.0446	0.103*	
H13B	0.1758	0.3257	0.0982	0.103*	
H13C	0.2254	0.2945	−0.0183	0.103*	
O4	0.41045 (9)	−0.08472 (18)	0.3880 (3)	0.0610 (6)	
C14	0.41311 (14)	−0.2269 (3)	0.4139 (5)	0.0673 (9)	
O5	0.40094 (15)	−0.2845 (3)	0.5499 (5)	0.1017 (10)	
C15	0.43333 (19)	−0.3023 (4)	0.2556 (6)	0.0896 (12)	
H15A	0.4749	−0.3250	0.2920	0.134*	
H15B	0.4272	−0.2414	0.1464	0.134*	
H15C	0.4109	−0.3889	0.2263	0.134*	

### Atomic displacement parameters (Å^2^)

**Table:**

	*U*^11^	*U*^22^	*U*^33^	*U*^12^	*U*^13^	*U*^23^
C1	0.0477 (15)	0.0635 (17)	0.0480 (15)	−0.0007 (12)	−0.0035 (12)	0.0094 (13)
C2	0.0505 (16)	0.0594 (17)	0.0648 (19)	0.0099 (13)	−0.0041 (14)	0.0092 (14)
C3	0.0501 (15)	0.0431 (12)	0.0586 (17)	0.0034 (11)	0.0097 (13)	0.0039 (13)
C4	0.0499 (15)	0.0457 (13)	0.0460 (15)	0.0032 (11)	0.0029 (12)	0.0039 (11)
C5	0.0389 (12)	0.0430 (12)	0.0428 (14)	−0.0014 (10)	0.0071 (11)	0.0087 (11)
C6	0.0471 (13)	0.0471 (13)	0.0435 (14)	0.0033 (10)	0.0081 (12)	0.0023 (11)
C7	0.0649 (18)	0.0655 (18)	0.0561 (17)	0.0190 (14)	0.0137 (16)	0.0037 (15)
C8	0.080 (2)	0.0593 (16)	0.0500 (16)	0.0057 (15)	0.0167 (16)	−0.0031 (14)
C9	0.0597 (16)	0.0617 (17)	0.0464 (16)	−0.0076 (13)	0.0107 (13)	−0.0014 (13)
O1	0.128 (2)	0.0996 (19)	0.0414 (12)	0.0212 (18)	0.0181 (13)	0.0080 (13)
C10	0.0410 (13)	0.0527 (14)	0.0430 (14)	−0.0023 (11)	0.0067 (11)	0.0048 (11)
C11	0.0475 (14)	0.0716 (18)	0.0660 (18)	−0.0132 (14)	0.0093 (13)	−0.0024 (16)
O2	0.0517 (10)	0.0500 (10)	0.0470 (10)	0.0037 (8)	−0.0007 (8)	0.0103 (9)
C12	0.0601 (16)	0.0520 (15)	0.0464 (16)	0.0186 (13)	0.0140 (14)	0.0063 (13)
O3	0.0948 (17)	0.0498 (11)	0.0581 (13)	0.0096 (11)	0.0132 (11)	0.0094 (10)
C13	0.079 (2)	0.0689 (19)	0.0520 (17)	0.0173 (16)	−0.0041 (15)	0.0027 (15)
O4	0.0668 (13)	0.0433 (10)	0.0724 (15)	0.0070 (8)	0.0127 (11)	0.0004 (9)
C14	0.0613 (18)	0.0466 (16)	0.085 (3)	0.0011 (13)	−0.0078 (17)	0.0000 (16)
O5	0.138 (3)	0.0503 (12)	0.117 (2)	−0.0015 (14)	0.024 (2)	0.0167 (14)
C15	0.093 (3)	0.0586 (18)	0.106 (3)	0.0127 (18)	−0.007 (2)	−0.0224 (19)

### Geometric parameters (Å, °)

**Table:**

C1—C2	1.524 (4)	C8—H8A	0.9700
C1—C10	1.528 (4)	C8—H8B	0.9700
C1—H1A	0.9700	C9—O1	1.209 (3)
C1—H1B	0.9700	C9—C10	1.526 (4)
C2—C3	1.512 (4)	C10—C11	1.543 (4)
C2—H2A	0.9700	C11—H11A	0.9600
C2—H2B	0.9700	C11—H11B	0.9600
C3—O4	1.453 (3)	C11—H11C	0.9600
C3—C4	1.523 (4)	O2—C12	1.346 (3)
C3—H3	0.9800	C12—O3	1.193 (4)
C4—C5	1.533 (3)	C12—O3	1.193 (4)
C4—H4B	0.9700	C12—C13	1.485 (4)
C4—H4A	0.9700	C13—H13A	0.9600
C5—C6	1.527 (3)	C13—H13B	0.9600
C5—C10	1.554 (3)	C13—H13C	0.9600
C5—H5	0.9800	O4—C14	1.340 (4)
C6—O2	1.452 (3)	C14—O5	1.200 (4)
C6—C7	1.523 (4)	C14—O5	1.200 (4)
C6—H6	0.9800	C14—C15	1.492 (5)
C7—C8	1.526 (4)	C15—H15A	0.9600
C7—H7B	0.9700	C15—H15B	0.9600
C7—H7A	0.9700	C15—H15C	0.9600
C8—C9	1.502 (4)		
			
C2—C1—C10	113.2 (2)	C7—C8—H8A	108.9
C2—C1—H1A	108.9	C9—C8—H8B	108.9
C10—C1—H1A	108.9	C7—C8—H8B	108.9
C2—C1—H1B	108.9	H8A—C8—H8B	107.7
C10—C1—H1B	108.9	O1—C9—C8	121.5 (3)
H1A—C1—H1B	107.8	O1—C9—C10	122.1 (3)
C3—C2—C1	110.9 (2)	C8—C9—C10	116.4 (2)
C3—C2—H2A	109.5	C9—C10—C1	110.4 (2)
C1—C2—H2A	109.5	C9—C10—C11	106.7 (2)
C3—C2—H2B	109.5	C1—C10—C11	110.3 (2)
C1—C2—H2B	109.5	C9—C10—C5	108.8 (2)
H2A—C2—H2B	108.1	C1—C10—C5	107.7 (2)
O4—C3—C2	111.8 (2)	C11—C10—C5	113.0 (2)
O4—C3—C4	105.9 (2)	C10—C11—H11A	109.5
C2—C3—C4	111.9 (2)	C10—C11—H11B	109.5
O4—C3—H3	109.1	H11A—C11—H11B	109.5
C2—C3—H3	109.1	C10—C11—H11C	109.5
C4—C3—H3	109.1	H11A—C11—H11C	109.5
C3—C4—C5	109.8 (2)	H11B—C11—H11C	109.5
C3—C4—H4B	109.7	C12—O2—C6	117.5 (2)
C5—C4—H4B	109.7	O3—C12—O2	123.5 (3)
C3—C4—H4A	109.7	O3—C12—O2	123.5 (3)
C5—C4—H4A	109.7	O3—C12—C13	126.0 (3)
H4B—C4—H4A	108.2	O3—C12—C13	126.0 (3)
C6—C5—C4	113.18 (19)	O2—C12—C13	110.4 (3)
C6—C5—C10	111.9 (2)	C12—C13—H13A	109.5
C4—C5—C10	111.34 (19)	C12—C13—H13B	109.5
C6—C5—H5	106.7	H13A—C13—H13B	109.5
C4—C5—H5	106.7	C12—C13—H13C	109.5
C10—C5—H5	106.7	H13A—C13—H13C	109.5
O2—C6—C7	109.1 (2)	H13B—C13—H13C	109.5
O2—C6—C5	105.94 (19)	C14—O4—C3	117.1 (3)
C7—C6—C5	110.1 (2)	O5—C14—O4	123.2 (3)
O2—C6—H6	110.5	O5—C14—O4	123.2 (3)
C7—C6—H6	110.5	O5—C14—C15	124.9 (3)
C5—C6—H6	110.5	O5—C14—C15	124.9 (3)
C6—C7—C8	111.7 (2)	O4—C14—C15	111.9 (3)
C6—C7—H7B	109.3	C14—C15—H15A	109.5
C8—C7—H7B	109.3	C14—C15—H15B	109.5
C6—C7—H7A	109.3	H15A—C15—H15B	109.5
C8—C7—H7A	109.3	C14—C15—H15C	109.5
H7B—C7—H7A	107.9	H15A—C15—H15C	109.5
C9—C8—C7	113.5 (3)	H15B—C15—H15C	109.5
C9—C8—H8A	108.9		

### Hydrogen-bond geometry (Å, °)

**Table:**

*D*—H···*A*	*D*—H	H···*A*	*D*···*A*	*D*—H···*A*
C7—H7A···O3	0.97	2.65	3.143 (4)	112
C8—H8B···O3^i^	0.97	2.62	3.551 (4)	161
C13—H13C···O3^ii^	0.96	2.63	3.533 (4)	156
C2—H2B···O5	0.97	2.65	3.134 (4)	111
C8—H8A···O5^iii^	0.97	2.44	3.309 (4)	149
C11—H11A···O5^iii^	0.96	2.70	3.662 (4)	178

Symmetry codes:  (i) *x*, *y*, *z*+1; (ii) −*x*+1/2, *y*−1/2, −*z*; (iii) *x*, *y*+1, *z*.
